# A simple method for establishing an ostrich model of femoral head osteonecrosis and collapse

**DOI:** 10.1186/s13018-015-0218-4

**Published:** 2015-05-21

**Authors:** Wenxue Jiang, Pengfei Wang, Yanlin Wan, Dasen Xin, Meng Fan

**Affiliations:** Department of Orthopedics, Tianjin First Center Hospital, 24 Fukang Rd. Nankai District, 300192 Tianjin, China

**Keywords:** Femoral head necrosis, Animal model, Ostrich, Liquid nitrogen

## Abstract

**Background:**

This study aimed to develop a simple method of creating an animal model of non-trauma femoral head osteonecrosis and collapse using African ostriches with weights similar to those of humans.

**Methods:**

Eighteen African ostriches were subjected to liquid nitrogen cryo-insult in the unilateral femoral head through surgical procedures using homemade cryogenic equipment combined with tract drilling inside the femoral head. Three animals were sacrificed at postoperative weeks 6 and 12, respectively, and the remaining animals were sacrificed at postoperative week 24. Bilateral femoral heads were harvested and subjected to gross observation, histological examination using hematoxylin and eosin staining, and radiographic examination. Micro-computed tomography was performed on a portion of the specimens at postoperative week 24, and angiographic examination of the femoral head was performed before sacrificing the animals.

**Results:**

Eight ostriches developed a limp at postoperative week 8, with a mean duration of 16.5 weeks. The postoperative femoral head specimens showed changes in contour and articular cartilage degeneration. Sagittal sectioning of the collapsed femoral head specimens revealed distinct boundaries among the osteonecrotic areas, osteosclerotic areas, and normal trabeculae. Histological examinations revealed active bone resorption in the osteonecrotic area of the subchondral bone, an increased number of fat cells, and active trabecular bone regeneration in the osteosclerotic areas. The postoperative radiographic examinations revealed that the height of the femoral head gradually decreased and progressed to collapse. Micro-computed tomography scans showed the interrupted trabecular bone with an irregular shape in the collapsed femoral head. Compared with the normal samples, angiographic findings revealed interrupted blood supply of the cryo-injured samples in some areas of the femoral heads, blood vessel narrowing, and decreased number of blood vessels in the cryo-injured areas.

**Conclusion:**

This study indicates that an animal model of osteonecrotic femoral head progressing to collapse can be established via a simplified method of cryosurgery. This model possesses histological features that are similar to those of humans; thus, it can be used as an ideal animal model for the study of femoral head necrosis.

## Introduction

The confirmed causes of osteonecrosis of the femoral head (ONFH) included corticosteroid using, excessive alcohol abuse, trauma, hemoglobinopathies, autoimmune diseases, and other relevant factors (smoking, hyperlipidemia), although the causes and mechanism of ONFH remained unknown [1, 2]. These factors induced the local ischemic within the femoral head by oneself or multi-factors combined and resulted in the bone cell apoptosis, consequently, caused the osteonecrosis and collapse. Surgeons suggested preserving the femoral head and arthroplasty for patients with the different pathological stage of ONFH. At the early stage of ONFH, especially in young patients, core decompression, core decompression combined with supplemental non-vascularized bone grafting, tantalum rods, rotational osteotomy, and vascularized bone grafting are common treatment options used by orthopedic surgeons in an effort to protect the subchondral bone, prevent bone collapse, and delay the need for artificial joint replacement [3, 4]. Therefore, the collapse of femoral head is known as the crisis to treat the ischemia and osteonecrosis of femoral head because the patients with a mild collapse of femoral head can even select the hip surface replacement. However, a widely accepted and effective therapeutic strategy to preserve the femoral head has not yet been developed. Thus, most patients will eventually require artificial joint replacement [5, 6].

Currently, the cause of traumatic ONFH has been identified; however, the pathophysiological mechanism of non-traumatic ONFH remains unknown and has become a widely researched field on ONFH models [7]. The ONFH animal models developed using rat, rabbit, sheep, pig, dog, and other quadrupedal mammals are usually successful at reproducing osteonecrosis, but they do not develop femoral head collapse [1, 8–11] due to the hindleg’s bearing. At present, the lack of animal models to mimic the complete process of human ONFH hinders the research into therapeutic strategies for this disease [12]. Therefore, establishing animal models that can mimic necrosis and collapse of femoral head will not only provide insight into the mechanism of ONFH but will also be of great significance to research efforts on the etiology and prevention of femoral head necrosis. Comparing with mammal of limbs weight-bearing, ostrich appears similar biomechanical structure of hip to human being [13, 14]. Therefore, ostrich, which weight is similar to human being, can be used to create the animal model to mimic the pathological feature of ONFH on normal weight-bearing. The ONFH model established by emu has proved that there were the similar pathological characteristics between emu and human being [15]. However, emu model cannot present the normal weight-bearing of human being due to the lower weight than human being. The aims of the present study were to simplify the preparation method of ONFH models and to mimic the similar pathological characteristics as human beings using African ostriches, the bipedal animals with weights similar to those of humans.

## Materials and methods

### Experimental animals and design

**Fig. 1:**
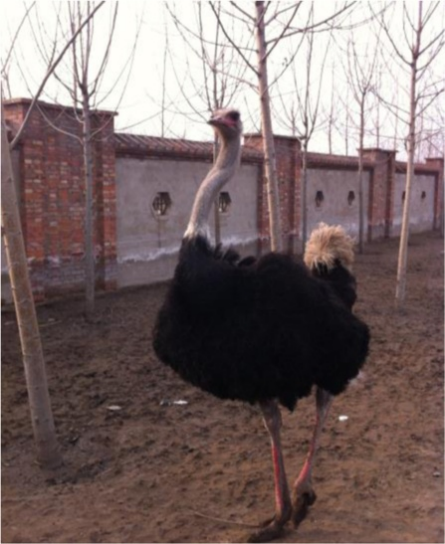
Experimental animal—Africa ostriches

Eighteen male healthy ostriches supplied by Tianjin Hongguang Farm, aged 1 year (Fig. 1), with a mean weight of 73.28 ± 3.72 kg (range, 67.41–79.15 kg) were used in the study. Six ostriches were reared in an area of 600 m^2^, and these animals were labeled with different numbers. All the animals were maintained on a 12-h light–dark cycle and received food and water ad libitum for 1 month without any limitations in their activities. Next, we randomly selected one side of the hip of the ostrich for surgery and considered the other side (normal hip) as a control. Three animals were randomly selected to be sacrificed at postoperative weeks 6 and 12, respectively, and the remaining animals were sacrificed at postoperative week 24.

The Animal Care and Use Committee of Tianjin Medical University approved the experimental protocol. The Principles of Experimental Animal Care, published by The Science and Technique Board of People’s Republic of China in 2006, were followed as were Chinese laws on animal protection, where applicable.

### Design of the liquid nitrogen cryogenic equipment

**Fig. 2:**
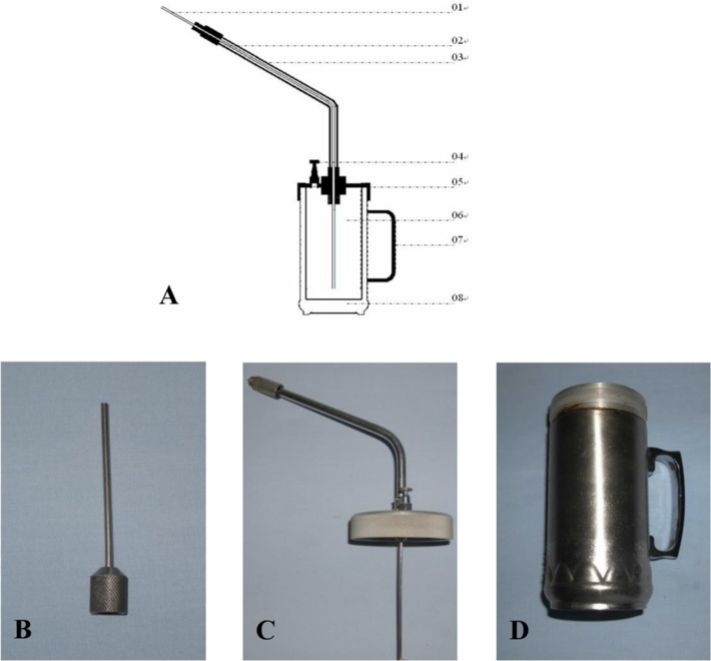
The experimental cryogenic equipment. **a** The sectional drawing of experimental cryogenic equipment. Component elements: *01* cryogenic probe, *02* liquid nitrogen transportation tube, *03* antifreezing tube, *04* pressure valve, *05* the cover of liquid nitrogen cavity, *06* the liquid nitrogen storage container, *07* hand handle, *08* thermal-protective coating. **b** A cryogenic probe. **c** A liquid nitrogen transportation system. **d** A liquid nitrogen storage system

Experimental cryogenic equipment was specifically designed for this study (Fig. 2a). The equipment was composed of a cryogenic probe (Fig. 2b), a liquid nitrogen transportation system (Fig. 2c), and a liquid nitrogen storage container (Fig. 2d). The operating mechanisms were as follows. The liquid nitrogen was stored in the container with a closed pressure valve. The pressure above the liquid in the container gradually increased because of the evaporation of the liquid nitrogen. Then, the liquid nitrogen was driven into the transportation tube and sprayed out through the probe.

### Surgical procedures

The ostriches were fasted 24 h prior to surgery. The procedure was initiated with anesthetic induction through an intramuscular injection of 8 mg/kg xylazine hydrochloride combined with 30 mg/kg ketamine hydrochloride; this was followed by anesthetic maintenance using an intravenous injection of 1 mg/mL ketamine hydrochloride saline at a speed of 4–6 drops/s via the jugular vein.

**Fig. 3:**
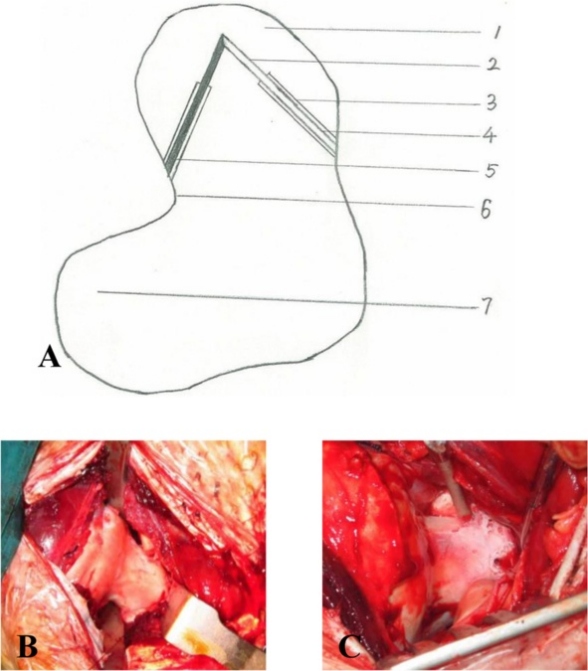
Surgical procedures. **a** Surgical procedures drawing: *1* Femoral head, *2* The freezing section of bone tunnel, *3* The protective section of bone tunnel, *4* The inserting side of aspirator, *5* The inserting side of cryogenic probe, *6* Femoral neck, *7* Femoral greater trochanter. **b** The greater trochanter. **c** The wan femoral head and femoral neck after being frozen

A skin incision was made on the left hip joint, with the center at the top of the greater trochanter and parallel to the longitudinal axis of the femur. Subsequently, the subcutaneous muscles were separated until reaching the hip joint capsule, which was then incised to expose the greater trochanter (Fig. 3b). Both sides of the femoral neck were exposed without hip dislocation. Using a 3-mm-diameter drill, a 3.5-cm-deep hole was drilled toward the subchondral tissue in the weight-bearing area of the femoral head from both the medial and lateral sides of the femoral head–neck junction. The interconnection between the drill access points on both sides was confirmed. A non-cryogenic bone protection area was created by drilling a 2-cm-deep tunnel along the original bone tunnel using a 5-mm-diameter hollow drill (Fig. 3a).

A 3-mm-diameter suction tube was inserted into the medial hole, and continuous suction was performed with a negative pressure of 0.02 MPa. The cryogenic probe containing 250 mL of liquid nitrogen was then inserted in the lateral hole. The peripheral soft tissue was protected from cryogen. The liquid nitrogen spray lasted for approximately 2 min (Fig. 3c), followed by a 3-min rewarming using 1000 mL of 0.9 % saline at 40 °C. The cycle of the cryo-insult-rewarming procedure was repeated three times. Finally, the surgical wound was rinsed, sutured, and covered with sterile gauze.

### Gross observation of the specimens

The femoral head contour, color, shape, and smoothness of the articular cartilage were grossly observed. A 5-mm-thick bone slice was harvested along the midline sagittal plane of the femoral head. The morphologies of the trabecular and subchondral bones were investigated.

### Histological examination

The 5-mm-thick bone slices were fixed in 10 % formalin solution for 72 h, followed by decalcification in neutral ethylenediaminetetraacetic acid solution for 3–4 weeks. They were then dehydrated, embedded in paraffin, and cut into 5-μm-thick slices for hematoxylin and eosin (H&E) staining. Trabecular bone alterations were observed under light microscopy.

### Imaging observation

Radiographic examination was performed using DR radiographic system (Siemens, Ysio, Germany). Anteroposterior radiographs of the same size were obtained for the bilateral femoral specimens of the ostriches. Micro-computed tomography (XM-Tracer 225, Union Air Science & Technique Limited Company, Beijing, China) was performed using the femoral heads of both sides.

### Angiographic examination

Two ostriches were selected randomly to perform the angiographic examination at 24 weeks postoperative. After inducing anesthesia in the ostriches, a midline abdominal incision was made to expose the abdominal aorta and inferior vena cava, which were then ligated and subjected to catheterization, respectively. Angiography-related solutions were injected from the abdominal aorta and ejected from the inferior vena cava. The procedure was initiated by sequential injection of 2000 mL of heparinized saline, 4000 mL of 0.9 % saline, and 4000 mL of 4 % formalin solution. A barium sulfate–gelatin suspension was prepared and injected rapidly with pressure until the white liquid overflowed. Next, both the inflow and outflow ends of the blood vessels were ligated and immobilized for 12 h. Specimens were collected once the gelatin had solidified, and a radiographic examination was performed to observe the blood vessels inside the femoral heads.

## Results

### Postoperative conditions

Among the 18 ostriches, 2 died from the anesthesia and 1 died from intestinal obstruction due to foreign body ingestion at postoperative week 8. The mean operation time was 130 ± 18.25 min. No wound infection or femoral neck fracture occurred in any of the animals after surgery. Three animals were randomly selected and sacrificed at postoperative weeks 6 and 12. The rest of the nine animals were sacrificed at postoperative week 24. No limp gait was observed in any of the animals at postoperative week 6. At postoperative week 12, one of the ostriches to be sacrificed was mildly lame. Among the ostriches that were sacrificed at postoperative week 24, seven were lame. The mean time to incapacitating lameness was 16.5 (±4.2) weeks after surgery. A limping gait was not obvious and was visible only during running at an early stage, but this progressed to a limping gait at postoperative weeks 3 and 4.

**Fig. 4:**
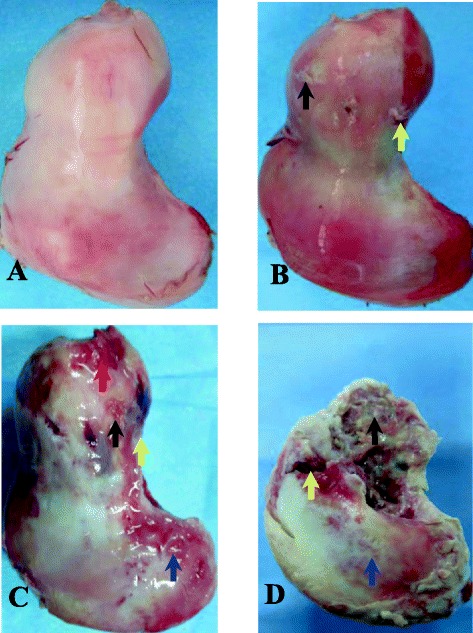
Postoperative conditions. **a** The normal ostrich femur head. **b** The ostrich femur head at postoperative week 6: the openings of the bone tunnel (*yellow arrow*). The focal cartilage exfoliation (*black arrow*). **c** The ostrich femur head at postoperative week 12: the changed contour of femoral head (*red arrow*). The covered openings of the bone tunnel (*yellow arrow*). Cartilage erosion (*black arrow*). The wrinkled cartilage at the side of trochanter (*blue arrow*). **d** The ostrich femur head at postoperative week 24: The complete collapsed femoral head (Fig. 4d, *black arrow*). The interrupted sclerotin of femoral neck (*yellow arrow*). The worn cartilage on the surface of the trochanter and the exposure of the subchondral bone (*blue arrow*)

As for the features of a normal ostrich femur, the cartilage on the femoral surface should be white with a smooth surface (without wrinkles, Fig. 4a). At postoperative week 6, the height of the femoral head did not decrease significantly compared with that of the control group (Fig. 4b). The color of the cartilage on the surface was dim. The openings of the drill tracts were still visible (yellow arrow). Focal cartilage exfoliation was observed (black arrow), though no alteration was observed in the cartilage on the surface of the trochanter. At postoperative week 12, we observed changes in the femoral head contour (Fig. 4c, red arrow), with the height being significantly lower than that on the control side. The openings of the bone tracts were covered by tissues (yellow arrow). Cartilage erosion occurred at the weight-bearing area (black arrow), and the cartilage at the trochanter became wrinkled (blue arrow). Complete loss of normal femoral contour occurred at postoperative week 24 (Fig. 4d, black arrow). The femoral neck was fractured due to alterations in the mechanical structure (yellow arrow). The cartilage on the surface of the trochanter was severely worn, resulting in the exposure of the subchondral bone (blue arrow).

**Fig. 5:**
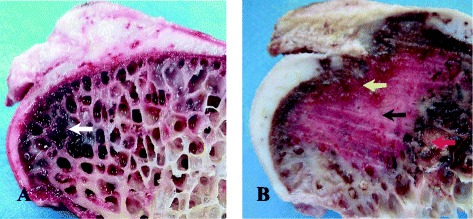
The comparing of normal and postoperative ostrich femur head. **a** The vertical plane of normal femoral head: the subchondral bone had no interruptions, and the bone marrow tissue was present within the bone trabecula (*white arrow*). **b** The vertical plane of collapsed femoral head postoperative: the subchondral bone was the *yellow arrow*, and the inflammatory pseudotumor the *red arrow*

In gross observation of normal femoral heads (Fig. 5a), in this study, we observed typical necrotic changes in the collapsed femoral head (Fig. 5b). In addition, the subchondral bone was interrupted and collapsed, the necrotic (yellow arrow) and sclerotic areas (black arrow) were visible in the femoral head, and the intertrabecular space narrowed or disappeared with the presence of an inflammatory pseudotumor (red arrow).

### H&E staining findings

**Fig. 6:**
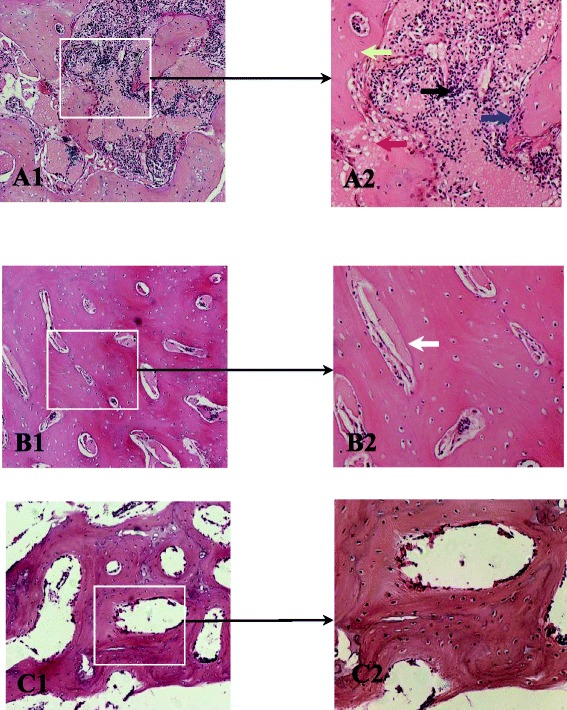
The result of H&E staining findings. **a** The H&E staining of the necrotic areas (*A1* by 100 light microscope, *A2* by 200 light microscope): the empty lacunae in the necrotic trabecular bone (*yellow arrow*). The irregular edges of the trabecular bone became due to bone resorption (*blue arrow*). The fibrous tissue within trabecular bone (*black arrow*) and fat cell (*red arrow*). **b** The sclerotic areas of trabecular bone (*B1* by 100 light microscope, *B2* by 200 light microscope): the trabecular bone thickened, the space of trabecular bone narrowed, and the nuclei of the active bone proliferation (*white arrow*). **c** The morphology of the normal trabecular bone: the uniform thickness and the space of trabecular bone and the normal morphology of bone marrow tissue

The trabecular bone tissue of the specimens harvested from the necrotic and sclerotic areas of the collapsed femoral heads, as well as the normal trabecular bone tissue, was subjected to H&E staining. The results showed the presence of empty lacunae in the trabecular bone in the necrotic area (Fig. 6a, yellow arrow). The edges of the trabecular bone became irregular (blue arrow) due to bone resorption. A large amount of fibrous tissue (black arrow) and fat cell (red arrow) proliferation occurred in the intertrabecular space. Within the sclerotic area (Fig. 6b), the trabecular bone thickened, the intertrabecular space narrowed (white arrow), and the nuclei of the new bone were strongly alkaline, indicating an active bone proliferation. In contrast, the normal trabeculae showed a continuous structure with smooth edges, and the normal bone marrow was observed in the intertrabecular space (Fig. 6c).

### Radiographic imaging findings

**Fig. 7:**
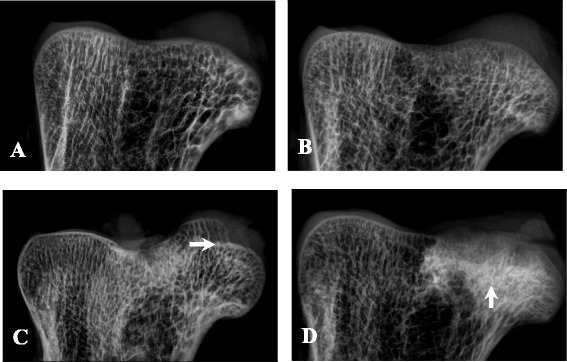
The radiographic imaging findings. **a** The X-ray imaging of normal ostrich femur head. **b** The X-ray imaging of ostrich femur head at postoperative week 6: no significant changes were observed in the femoral head contour or bone density. **c** The X-ray imaging of ostrich femur head at postoperative week 12: alterations in the femoral head contour (*arrow*) and decrease in the height of the femoral head. **d** The X-ray imaging of the collapsed ostrich femur head: the collapsed femoral head and high-density shadows at the center of the femoral head (*arrow*)

The radiographs of the normal femoral heads demonstrated an intact shape of the femoral heads, a continuous structure of the subchondral bones, regular morphology of the trabecular bone, and uniform density within the femoral head (Fig. 7a). At postoperative week 6, no significant changes were observed in the femoral head contour or bone density (Fig. 7b). At postoperative week 12, alterations in the femoral head contour were observed, with a decrease in the height of the femoral head (Fig. 7c). At postoperative week 24, the femoral head contour apparently disappeared, the bone collapsed at the weight-bearing area of the femoral head, and high-density shadows were observed at the center of the femoral head (Fig. 7d).

### Micro-CT imaging findings

**Fig. 8:**
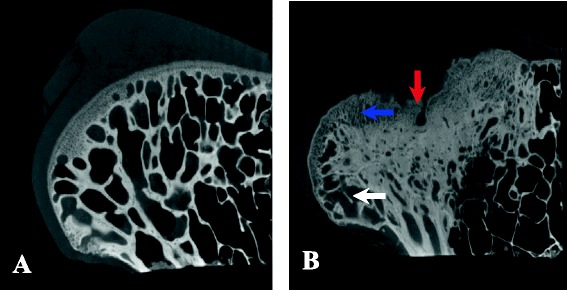
The micro-CT imaging findings. **a** The micro-CT images of the normal femoral heads: a continuous subchondral bone and a regular arranged trabecular bone. **b** The micro-CT images of the collapsed femoral heads at 24 weeks postoperative: the interrupted subchondral bone (*red arrow*), decreased bone density (*blue arrow*), and the interrupted trabecular bone (*white arrow*)

Micro-CT images of the femoral heads from the control group showed a continuous structure of the subchondral bone tissue, an intact contour of the femoral head, and a regular arrangement of the trabecular bone tissue with consistent morphology and evenly distributed intertrabecular space (Fig. 8a). Images of the collapsed femoral heads (Fig. 8b) demonstrated an interrupted structure of the subchondral bone tissue (red arrow) and the presence of necrotic lesions or osteoporosis in the femoral head, with significantly lower density than the normal controls (blue arrow). Moreover, the trabeculae were thin and partially interrupted (white arrow), the trabeculae beneath the necrotic area showed an irregular arrangement, and the narrowed intertrabecular space was filled with shadows at an abnormal density.

### Angiographic findings

**Fig. 9:**
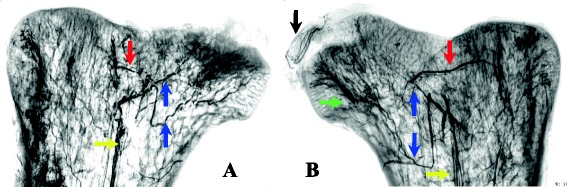
The angiographic findings of bilateral ostrich femoral heads at postoperative week 6. **a** The operation side. **b** The control side

Angiograms of the normal ostrich femoral heads demonstrated three major blood vascular supplies (Fig. 9b). First, a trifurcation of the intramedullary nutrient artery (yellow arrow) was observed at the base of the femoral neck, with two branches supplying the femoral head (blue arrow) and another one supplying the trochanter (red arrow). Second, a blood vessel was observed within the round ligament, which is similar to the foveolar artery in humans (black arrow). Third, numerous perforating arteries in the periosteum of the femoral neck entered vertically at the femoral head–neck junction and traveled in the femoral head (green arrow). Angiograms of the collapsed femoral head (Fig. 9a) demonstrated that the intramedullary nutrient artery traveled tortuously at the base of the femoral neck, and the arterial branches narrowed and terminated in the sclerotic area (blue arrow). The blood vessels within the round ligament were not visible, and the blood supply was interrupted. The number of perforating vessels surrounding the femoral neck was reduced, and compared to the control group, these vessels exhibited an obscure morphology.

## Discussion

Femoral head collapse is a characteristic change during the mid-late stage of ONFH and is used as a standard feature to evaluate the successful establishment of an ONFH animal model. Conzemius successfully established an emu model of femoral head collapse using liquid nitrogen cryo-insult [16]. Some researchers have conducted gait analyses of emus and have confirmed that the mechanical environment within the emu hip is similar to that of human beings [13, 14]. In the present study, we used African ostriches with weights similar to humans; with this model, we were able to verify limping gaits and femoral head collapse, indicating the superiority of using bipedal animals in establishing an ONFH model [15]. Studies have also shown that body weight is associated with femoral head necrosis [17]. In the study by Conzemius, limp occurred at an average time point 11.75 weeks postoperative in emu models [16]. In this study, limp did not occur sooner (at an average time point 16.5 weeks postoperative) despite the heavier weights of the subjects, which might be related to differences in the extent of bone damage, animal species, and standards for limp identification.

In studies of ONFH models, the method used to selectively create focal subchondral necrosis of the femoral head is the key. To pursue this goal, designing a navigation device inside the bone has become a new direction of study. Reed et al. designed a cryoprobe, which was placed under radiographic guidance into the subchondral bones to create necrosis of the target area [18]. Goetz performed a finite element analysis on the necrotic area induced by this device and confirmed the reliability of this technique [19, 20]. Fan established an ONFH model using a more advanced cryo-heating probe [15]. However, employment of this equipment limited the application of this technology in establishing ONFH models because of the size and cost. In this study, we developed more compact liquid nitrogen cryogenic equipment. The area of cryo-insult was restricted by controlling the liquid nitrogen volume. Furthermore, possible damage due to contact of the cryoprobe with non-targeted bones at the femoral head was prevented by expanding the drilling size through the bony tract, thereby restricting cryo-insult to only the subchondral bone. Precaution should be taken to insert a suction tube prior to the modeling procedure in an attempt to remove the blood inside the drilling tract, thus preventing tract obstruction caused by frozen blood during the cryogenic process. During the modeling procedure, only the joint capsule was incised, thereby avoiding severance of the round ligament and joint dislocation; therefore, we were able to maintain stability of the hip joint to the maximum extent. In this study, we verified that the ostriches could completely wake up and achieve weight-bearing standing 3 h after surgery. No postoperative complications of hip dislocation occurred in any of the animals.

The opening of the bony tract was still visible during the gross observation of the femoral specimen at postoperative week 6, and it was filled with tissues at postoperative week 12. No significant bone defects were observed on the radiographic images of the femoral heads. Meanwhile, micro-CT imaging findings confirmed that the collapse only occurred in the weight-bearing area of the femoral heads. Hence, we believe that the drill tracts in the femoral head did not change the mechanical structure of the ostrich femur, nor were they the direct cause of femoral head collapse.

Results of the postoperative gross observation, imaging examination, and histological examination indicated that the ONFH model established using the proposed method created human-like pathological changes. Gross observation of the sagittal section of the specimens revealed typical osteonecrosis and osteohyperplasia along with the presence of inflammatory pseudotumor, which is consistent with the H&E staining findings. However, inconsistent with the H&E staining results of the normal femoral heads, a large number of fat cells were present at the intertrabecular space in the necrotic area of the collapsed femoral head specimens, which is usually a typical feature of a hormone-induced ONFH model. This result suggests that fat cell proliferation likely represents the natural progression of ONFH rather than a specific feature of a particular type of ONFH.

Nevertheless, this study had significant limitations. First, performing surgeries under radiographic guidance and magnetic resonance imaging examinations was not possible in the African ostriches because of the large body size of the animals. Therefore, to determine the appropriate drilling depth, technique, and position to ensure interconnection between the tracts on both sides, sufficient preoperative practice using normal ostrich femoral head samples is required. In vitro preliminary experiments have shown that 250 mL of liquid nitrogen cryo-insult could result in a 3-cm-diameter spherical bone injury at ostriches’ femoral head. However, the extent of the damage will diminish during the establishment of the model because of the influence of the animal’s body temperature and blood flow. In addition, ostriches might not be the most appropriate study subjects because of the high costs of feeding and rearing, their large body size, and the nature of the species, which differs from that of mammals [18, 21].

This study demonstrates that weight-bearing of the hip joints and localization of subchondral bone injury is critical for the establishment of animal ONFH models. Simplifying the required equipment and including smaller experimental animals will be the focus of future research for the development of ONFH models [22].

## Conclusions

This study indicates that an animal model of non-trauma osteonecrotic femoral head progressing to collapse can be established via a simplified method of cryosurgery. This model possesses histological features that are similar to those of humans; thus, it can be used as an ideal animal model for the study of femoral head necrosis.
